# Rotational symmetry of photonic bound states in the continuum

**DOI:** 10.1038/s41598-020-75308-x

**Published:** 2020-10-26

**Authors:** Liangsheng Li, Yunzhou Li, Yong Zhu, Hongcheng Yin

**Affiliations:** 1 Science and Technology on Electromagnetic Scattering Laboratory, Beijing, 100854 China; 2 Kunming Shipbuilding Equipment Research and Test Center, Kunming, 650051 China

**Keywords:** Metamaterials, Photonic crystals

## Abstract

The bound states in the continuum (BICs) have been investigated by simulating the optical reflectivity of a tri-layer photonic crystal slab. We found that optical BICs can occur in a class of photonic crystal systems with $c_{1}^{z}$, $c_{2}^{z}$ or $c_{4}^{z}$ rotational symmetries, which are constructed by three identical photonic crystal slabs. By applying the two mode coupled model, we obtain the reflectivity formula to fit the numerical data and evaluate the lifetime of radiation decay. In vicinity of BIC, the lifetime diverges as a power law form, when approaching the BIC point. The infinity life time of $c_{1}^{z} {\text{ } - \text{ BIC}}$ in the tri-layer structure indicate that it is a true BIC. The $c_{1}^{z} {\text{ } - \text{ BIC}}$ occurs robustly in tri-layer structures, but the resonance frequency of the BICs is dependent on the permittivity of slab, air-hole size and hole shape.

## Introduction

Bound states in the continuum (BICs) were firstly predicted in quantum systems by von Neumann and Wigner^1^, and were extensively observed in various wave physics^2^. Optical BICs have been deeply investigated by simulations and experiments on photonic lattices^2–22^, waveguides^23–26^, compact structures^27–31^, and metasurfaces^32–34^. A true BIC can be understood as the condition that the resonance width vanishes and leakage is forbidden^2^, i.e., eigenmodes have infinite both qualify factors and lifetime. The BICs can be easily found in zero permittivity or permeability media, when the complex eigenfrequency becomes purely real^27–30^. On the other hand, the BIC could also occur in photonic crystal (PhC) systems with ideal lossless infinite structures^6,7^. Recently, the BICs in PhC slabs are related to topological charges, restricted by in-plane point-group symmetries of the system^9,35^. However, when the in-plane symmetry of structure is broken and become $c_{1}^{z}$ symmetry (identity), the systems can also support high-quality factor resonances^34^. This type of BIC can be realized as a quasi-BIC, where both the life time and resonance width become finite. The quasi-BICs in metasurfaces by changing the in-plane symmetry display extremely high conversion efficiency for the third harmonic generation^36^.

In the vicinity of the BIC, these are a series of resonant modes, where the quality factor of modes is still very high. These resonant modes, in the near-BIC regime, are similar to quasi-BIC and can be excited by free propagating plane waves. The mode with a high quality factor in a large region of parameter space is of practical importance for building fabricated devices, such as BIC–laser^10^ and optical resonators^37^. Some BICs show high quality factor resonances for a very large range of wave vector, because the quality factors of the resonant modes satisfy an inverse fourth power law relation^18^. The wide parameter range high quality factor resonances of BIC play an importance role in device design as fabrication tolerances. The investigations of BICs stimulate the significant progress of polarization vortices^38,39^.

## Results

### BICs and rotational symmetry

**Figure 1:**
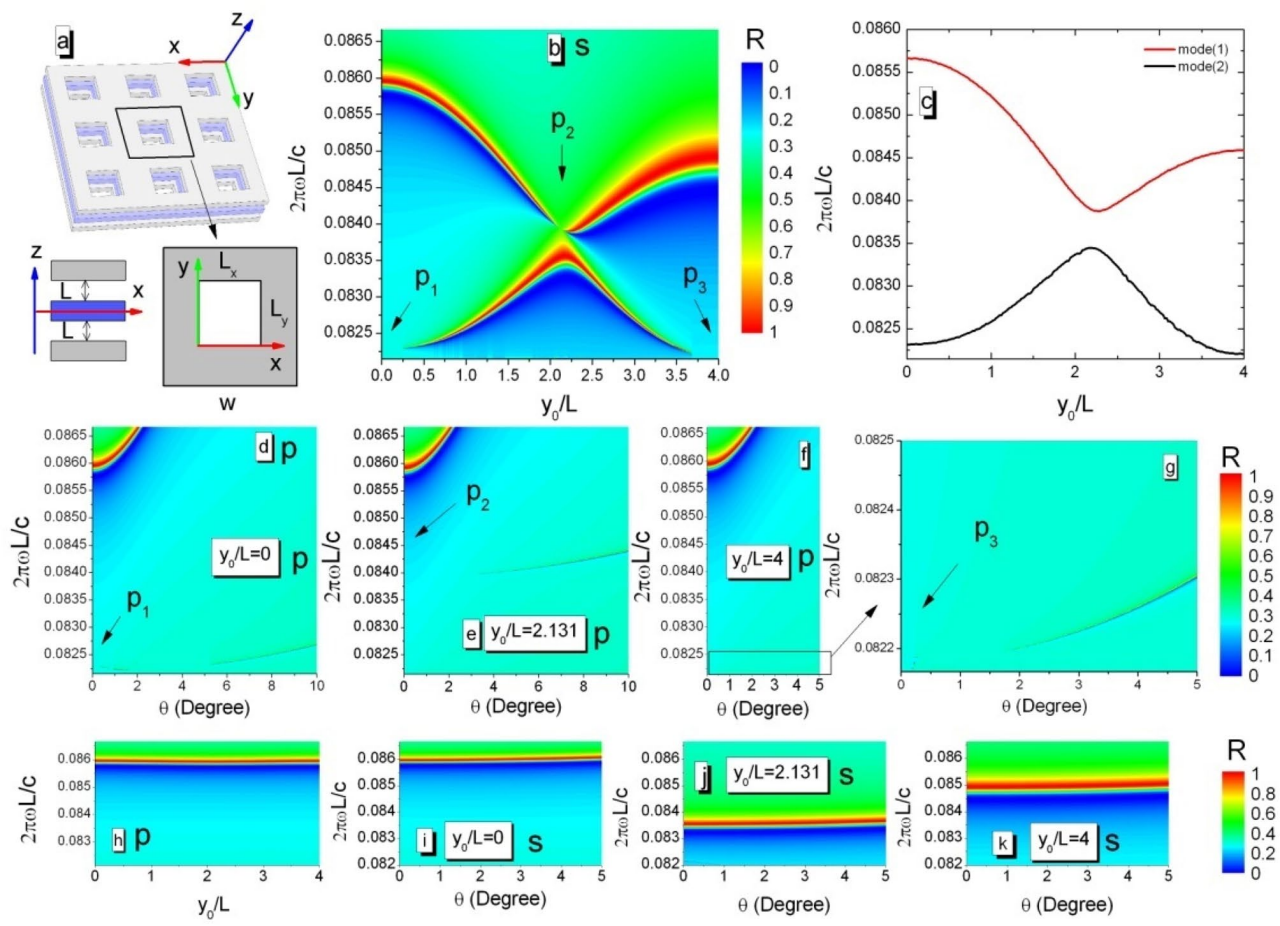
(**a**) Schematic of a tunable tri-layer structure consisting of parallel PhC slabs. It is periodic in the x and y direction. The interspaces between PhC slabs are identical to ensure the up–down mirror symmetry in the z direction. The middle layer is able to move along the y-axis. (**b**) Numerical reflectivity spectra (R) at normal incidence (s-polarized) as a function of incident frequency and displacement of the middle layer. P-Numbers within the contour plot indicate the BICs where Fano features disappear. (**c**) Calculated eigen-frequeucy for Γ point of the first Brillouin zone versus displacement of the middle layer. Reflectivity spectra (p-polarized) as a function of incident angle and frequency for the various value of the displacement (**d**) $y_{0} /L = 0$, (**e**) $y_{0} /L = 2.131$, (**f**),(**g**) $y_{0} /L = 4$. (**h**) R at normal incidence (p-polarized) versus frequency and displacement. (**i**)–(**k**), for the various displacement, R (s-polarized) versus incident angle and frequency.

We show that a true BIC, with vanishing resonance width, can occur in broken in-plane symmetry PhC structures. Consider a mechanically tunable tri-layer structure consisting of three same PhC slabs, where the middle slab (blue) is able to move along the y-axis, as shown in Fig. 1a. The full wave simulation uses CST software. The single PhC slab with finite thickness L has a square lattice (periodicity W) of square air holes ($L_{x} = L_{y}$) and $c_{4}^{z}$ rotational symmetry. Here, $c_{4}^{z}$ means ${90}^{{\text{o}}}$ rotation around z-axis. The interspaces between near-nearest slabs are identical to keep the mirror symmetry in the z direction. The tri-layer structure has time-reversal symmetry $\varepsilon \left( {\vec{r}} \right) = \varepsilon^{*} \left( {\vec{r}} \right)$, where $\varepsilon$ is permittivity of the slab and $*$ is the complex conjugate operator. Although the middle slab can move along the y-axis, the tri-layer structure always keep mirror symmetry $\varepsilon (x,y,z) = \varepsilon (x,y, - z)$.

Without loss of generality, we take the dielectric constant of the slab $\varepsilon = 4.5$, hole sizes $L_{x} = L_{y} = 2.4L$, interspaces ${\text{D}} = L$, and periodicity $W = 8L$. When light normally incident on the tri-layer PhC structure, Fano resonances should be observed in the reflectivity spectra, for the s-polarized mode (the electric field along the x axis), shown in Fig. 1b. Those resonances are characterized by the asymmetric profile consisted of blue and red branches corresponding to the reflectivity of zero and one, respectively. For various displacements ($y_{0}$) of the middle layer, the tri-layer structures exhibit different waveguide modes as shown in Fig. 1c. The anticrossing behavior of modes indicates that the BIC at P_2_ is a Friedrich–Wintgen type BIC^40^. Because Fano resonances originate from the interference between out-of-plane far-field radiation and in-plane waveguide modes, the resonance profiles can be changed by moving the middle layer along the y direction. In this tri-layer structure as mirrors to form a tunable optical cavity, the interference is highly sensitive to $y_{0}$. When the displacement is suitable at the correct phase matching conditions, the blue and red branches should meet each other. Then, a BIC occurs. The y_0_-dependence interference indicates that the Fabry–Perot-type resonance plays an important role in the formation of BICs in the tri-layer structure. By continuously changing $y_{0}$, we find three BICs guided by the arrows, as shown in Fig. 1b. BICs at P_1_, P_2_, and P_3_ can also be observed for the p-polarized mode (the electric field along the y axis) by changing the incident angle ($\theta$) shown in Fig. 1d–g. However, when s-polarized light normally incident on slabs, Fano resonances with finite resonant width are observed around the reduced frequency (0.086) and insensitive to the displacement as shown in Fig. 1f. By changing the incident angle, the tri-layer structures exhibit that, for the p-polarized mode, fano resonances still remain the finite resonant width shown in Fig. 1i–k.

**Figure 2:**
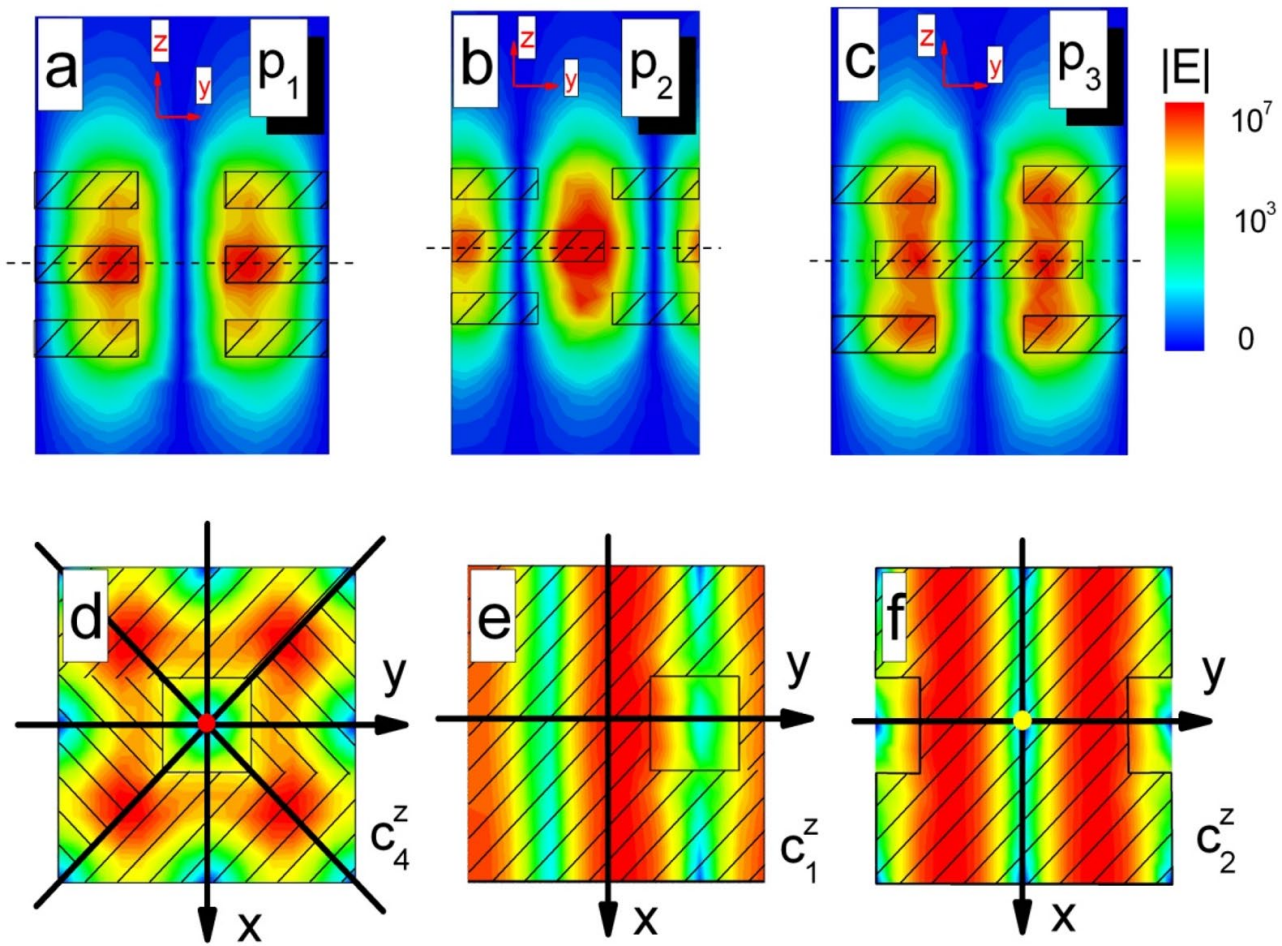
(**a**–**c**) The electric field intensities at P_1_, P_2_ and P_3_ are presented as functions of the y(z) coordinate parallel (perpendicular) to the slabs. (**d**–**f**) the electric field intensities in xy-plane at P_1_, P_2_ and P_3_ have $c_{4}^{z}$, $c_{1}^{z}$ and $c_{2}^{z}$ symmetries, respectively. The red (**a**) and yellow (**c**) dots mark the $c_{4}^{z}$ and $c_{2}^{z}$ symmetry axes, respectively.

In order to gain further physical insights of the BIC in the tri-layer structure, we plot the distributions of the electric field intensity in yz-plane and xy-plane at BICs in Fig. 2. We clearly see that the field distributions become localization inside the structure, while the outside field tends to vanish. Because the tri-layer structure always contains a mirror symmetry for different displacements in the z direction, the electric field intensities also have the mirror symmetry as shown in Fig. 2a–c. When $y_{0} = 0$, the structure has $c_{4}^{z}$ rotational symmetry $\varepsilon \left( {x,y,z} \right) = \varepsilon \left( { - y,x,z} \right)$. The electric field intensity also has $c_{4}^{z}$ rotational symmetry as shown in Fig. 2d. Then, we have $\varepsilon \left( {\vec{r}} \right) = \varepsilon \left( {R_{4} \vec{r}} \right)$ where the operator $R_{n}$ rotates vectors by an angle ${2}\pi {\text{/n}}$ about the z-axis. The appearance of a BIC may be understood as the geometric symmetry forbids coupling to any far-field radiation. The electric field of the resonance can be $\vec{E} = e^{{i\vec{k} \cdot \vec{r}}} \vec{u}\left( {\vec{r},\vec{k}} \right)$ by using Bloch’s theorem, where $\vec{k} = \left( {k_{x} ,k_{y} ,0} \right)$ and $\vec{u}$ is periodic function in $(x,y)$^41^. Then, we could use the vector field rotator ${\text{O}}\left( {R_{4} } \right)$ to rotate the electric fields $\vec{E}$ as ${\text{O}}\left( {R_{4} } \right) \cdot \vec{E}\left( {\vec{r}} \right){ = }R_{4} \vec{E}\left( {R_{{4}}^{ - 1} \vec{r}} \right)$. Because the structure is invariant under transformation $R_{{4}}$, the electric fields $R_{4} \vec{E}\left( {R_{{4}}^{ - 1} \vec{r}} \right)$ are also the solutions of Maxwell’s equations with the same resonance frequency of $\vec{E}$, but they could differ by a phase factor $\vec{E}{ = }e^{{i\theta_{4} }} R_{4} \vec{E}\left( {R_{{4}}^{ - 1} \vec{r}} \right)$. If applying the rotational transformation 4-times, we can return to the original electric fields.

When moving the middle layer, $c_{4}^{z}$ symmetry of tri-layer structure is broken and $c_{4}^{z} {\text{ } - \text{ BIC}}$ disappears. However, when $y_{0} /L = {2}.{131}$, the accidental phase matching condition is satisfied to cancel the radiation mode and the $c_{{1}}^{z} {\text{ } - \text{ BIC}}$ occurs. This BIC arises from the interaction of two modes^42^.This tri-layer structure only has $c_{1}^{z}$ rotational symmetry shown in Fig. 2b, e, which means BICs can be found in the multi-layer structures without $c_{2}^{z}$ rotational symmetry. Here, $c_{1}^{z}$ means 360° rotation around z-axis. It is noted that the tri-layer structure always keeps the time reverse symmetry and the mirror symmetry for the any displacements of the middle layer.

When $y_{0} /L = 4$, the symmetry-protected BIC appears since the tri-layer structure recovers $c_{2}^{z}$ rotational symmetry $\varepsilon \left( {x,y,z} \right) = \varepsilon \left( { - x, - y,z} \right)$ as shown in Fig. 2c,f. Then, we have $\vec{E}{ = }e^{{i\theta_{{2}} }} R_{{2}} \vec{E}\left( {R_{{2}}^{ - 1} \vec{r}} \right)$. If we rotate the system twice, the electric fields add another phase factor $\vec{E}{ = }e^{{i{2}\theta_{{2}} }} \vec{E}$, so the phase $\theta_{{2}}$ can only take on values of ${0}$ or $\pi$. In fact, the displacements of middle layer, that break rotational symmetry, reduce the infinite lifetime of BICs but add a tuning parameter of resonant state. As a result, optical BICs can occur in the tri-layer photonic crystal systems with $c_{1}^{z}$,$c_{2}^{z}$ or $c_{4}^{z}$ rotational symmetries. However, the distributions of the electric field intensity at BICs are sensitive to the rotational symmetry.

At BICs, the lifetime of radiation decay becomes infinity. Thus, we might apply the temporal coupled mode theory to estimate the lifetime from the numerical data. Here, we consider a system that possesses two modes coupling with each other, and the non-Hermitian Hamiltonian of the tri-layer structure is

1 $$H = \Omega - i{\rm P} = \left( {\begin{array}{*{20}c} {f_{1} } & {} \\ {} & {f_{2} } \\ \end{array} } \right) - i\left( {\begin{array}{*{20}c} {\gamma_{1} } & {\sqrt {\gamma_{1} \gamma_{2} } } \\ {\sqrt {\gamma_{1} \gamma_{2} } } & {\gamma_{2} } \\ \end{array} } \right)$$

Here, $f_{1}$ and $f_{2}$ are the resonance frequencies of the resonators. The radiative-decay lifetime can be defined by $\tau_{i} = 1/\gamma_{j}$. Due to the energy conservation and time-reversal symmetry^43–45^, the reflectivity can be easily obtained and given by

2 $$R\left( \omega \right) = \left| {\frac{{s_{1}^{out} }}{{s_{1}^{in} }}} \right|^{2} = \left| {\frac{{\lambda_{1} \cos \theta - i\lambda_{2} \sin \theta }}{{\lambda_{1} + i\lambda_{2} }}} \right|^{2}$$

where

3 $$\begin{gathered} \lambda_{1} = \left( {f - f_{1} } \right)\left( {f - f_{2} } \right) \hfill \\ \lambda_{2} = \gamma_{2} \left( {f - f_{1} } \right) + \gamma_{1} \left( {f - f_{2} } \right) \hfill \\ \end{gathered}$$

Here, $f_{1}$ and $f_{2}$ are the resonance frequencies of the resonators. The radiative-decay lifetime can be defined by $\tau_{i} = 1/\gamma_{j}$.

**Figure 3:**
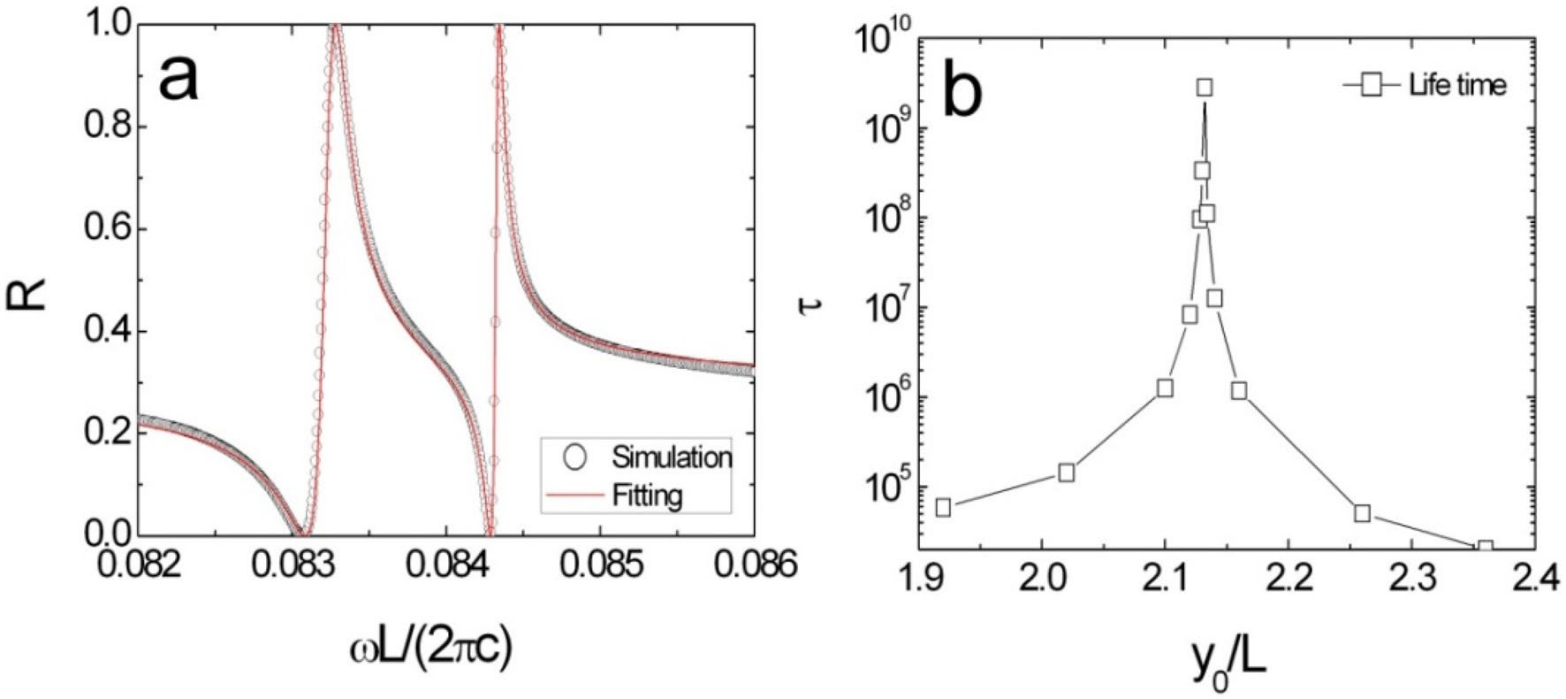
(**a**) Reflectivity spectrum upon normally incident wave. Solid line is the analytic expression obtained from Eq. (1) and the open circles are the simulation results. (**b**) The life time as a function of the displacement near $c_{{1}}^{z} {\text{ } - \text{ BIC}}$.

Then, we could apply Eq. (2) to fit the numerical reflectivity as shown in Fig. 3a. From the fitting curves, the resonance frequencies and the lifetimes might be estimated for various $y_{0}$ values. The lifetime of the resonance goes to infinity at $c_{{1}}^{z} {\text{ } - \text{ BIC}}$ shown in Fig. 3b. In vicinity of BIC, the lifetime can be written into a power law form as

4 $$\tau \propto \left\{ {\begin{array}{*{20}l} {\left( {y_{0} - y_{0}^{BIC} } \right)^{{ - \alpha^{P} }} } \hfill & {y_{0} > y_{0}^{BIC} } \hfill \\ {\left( {y_{0}^{BIC} - y_{0} } \right)^{{ - \alpha^{N} }} } \hfill & {y_{0} < y_{0}^{BIC} } \hfill \\ \end{array} } \right.$$

where $y_{0}^{BIC}$ is the displacement for the BIC situation. $\alpha^{{N{/}P}}$ is the exponent of power law and a positive real number. Naturally, $\tau$ become infinity, when $y_{0} { = }y_{0}^{BIC}$, and a BIC occurs.

**Figure 4:**
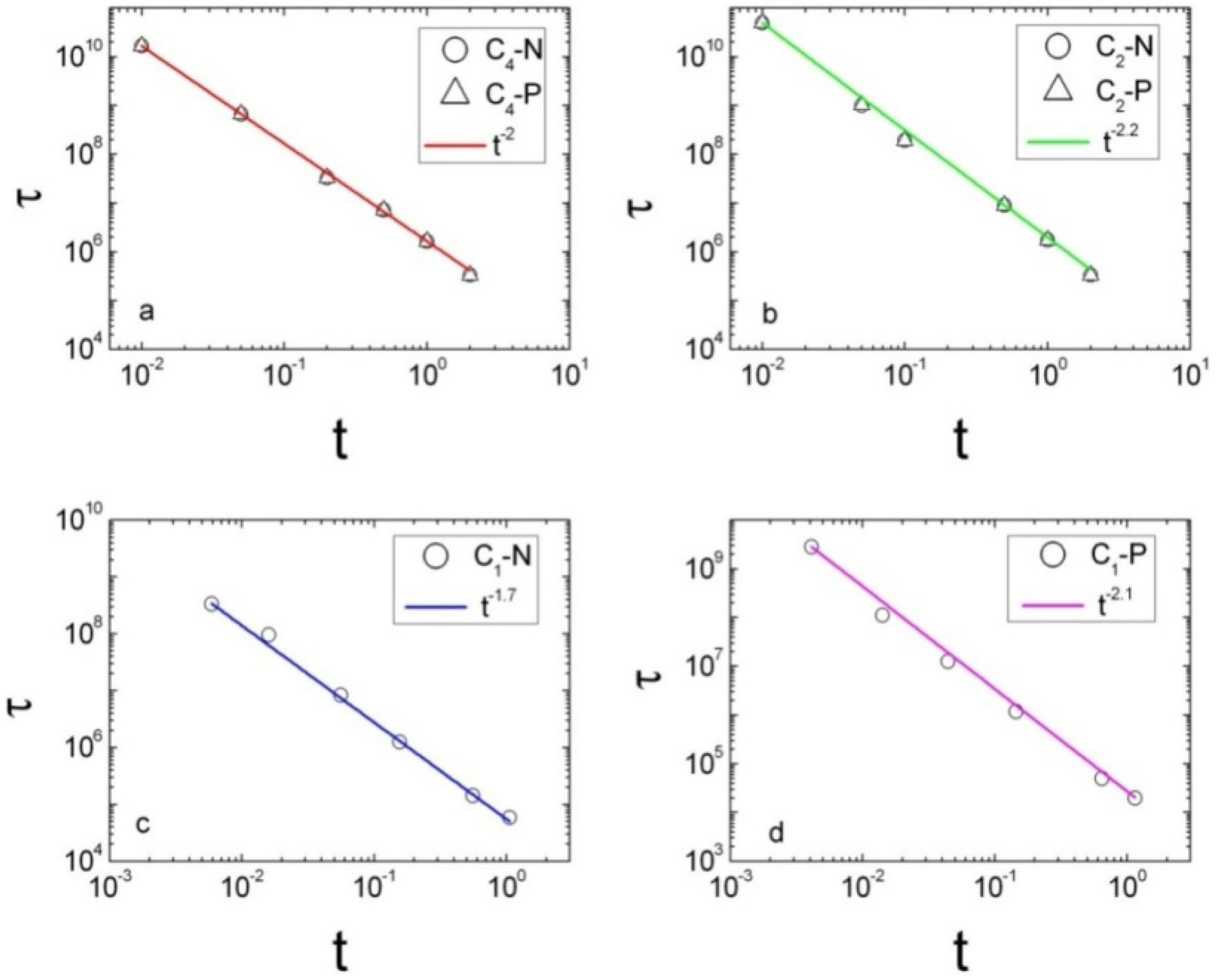
Log–log plot of lifetime versus the dimensionless distance. (**a**) For $c_{4}^{z} {\text{ } - \text{ BIC}}$, we obtain the exponents $\alpha_{4}^{N} = \alpha_{4}^{P} = 2$. (**b**) For $c_{2}^{z} {\text{ } - \text{ BIC}}$, the exponents become $\alpha_{2}^{N} = \alpha_{2}^{P} = 2.2$. For $c_{1}^{z} {\text{ } - \text{ BIC}}$, (**c**) the exponent in the negative region of the dimensionless distance $\alpha_{1}^{N} = 1.7$ is estimated, and (**d**) in the positive region the exponent is $\alpha_{1}^{P} = 2.{1}$.

Furthermore, the exponents of lifetime can be estimated by the power law relations shown in Fig. 4. Here, we define the dimensionless parameter $t = \left| {y_{0} - y_{0}^{BIC} } \right|/L$. The Log–Log plots of $\tau$ versus $t$ become straight lines with slopes $\alpha^{P}$ and $\alpha^{N}$. For $c_{4}^{z} {\text{ } - \text{ BIC}}$, where the tri-layer structure has $c_{4}^{z}$ rotational symmetry, two exponents of lifetime are identical with each other. When the structure is changed and has $c_{{2}}^{z}$ rotational symmetry, the exponents are still same with each other, but the values become larger. For $c_{1}^{z} {\text{ } - \text{ BIC}}$, the estimated exponent is $\alpha_{1}^{N} = 1.7$ in the negative region, and the exponent is $\alpha_{1}^{P} = 2.{1}$ in the positive region.

### Size and shape of holes

**Figure 5:**
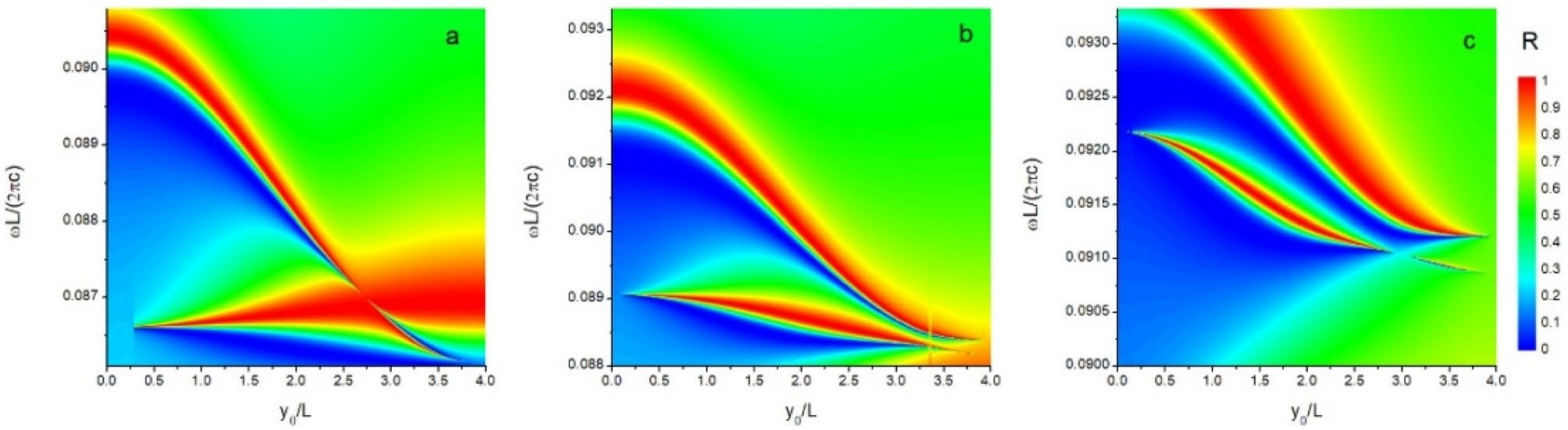
Reflectivity spectra of normal incident light as a function of incident frequency and displacement of the middle layer for the various sizes of square air-hole: (**a**) $L_{x} = 3.6L$, (**b**) $L_{x} = 4L$, and (**c**) $L_{x} = 4.4L$.

In order to examine the robustness of the relations between exponents, the size of square air hole in the slabs is enlarged, and the reflectivity spectra are plotted in Fig. 5. Then, the lifetime can be extracted by Eq. (4), and the exponents of lifetime are also estimated by the power law form. If the tri-layer structure has $c_{4}^{z}$ rotational symmetry, two exponents of lifetime keep a constant value ($\alpha_{4}^{{N{\text{/P}}}} = 2$) for various air hole sizes. The integer form of exponents indicate that the $c_{4}^{z} {\text{ } - \text{ BIC}}$ can be understood by a topological defect vortex^9,35^. Then, the tri-layer structure, for various air hole sizes, can be changed to produce $c_{2}^{z} {\text{ } - \text{ BIC}}$, it is found that two exponents ($\alpha_{2}^{N/P}$) still keep identical with each other. When increasing the air hole size, the displacement $y_{0}^{BIC}$ of $c_{1}^{z} {\text{ } - \text{ BIC}}$ becomes larger. It is noted that there is a transition point $R_{t} { = 4}$, where $c_{1}^{z} {\text{ } - \text{ BIC}}$ do not be observed. This accidental disappearance of $c_{1}^{z} {\text{ } - \text{ BIC}}$ might originate from the specially required displacement $y_{0}^{BIC} = 4$ of $c_{1}^{z} {\text{ } - \text{ BIC}}$. When $L_{x} /L \ge R_{t}$, two $c_{2}^{z} {\text{ } - \text{ BICs}}$ appear. When $L_{x} /L = 4.4$, for the higher frequency $c_{2}^{z} {\text{ } - \text{ BIC}}$ ($\omega L/2\pi c = 0.0{912}$), the exponent is $\alpha_{2}^{{N{/}P}} = {2}{\text{.0}}$, but for the lower frequency case ($\omega L/2\pi c = 0.0{908}$), the exponent becomes $\alpha_{2}^{N/P} = 2.1$. Furthermore, the $c_{1}^{z} {\text{ } - \text{ BIC}}$ can appear at various air hole size except for the transition point. When $L_{x} /L < R_{t}$, the $c_{1}^{z} {\text{ } - \text{ BIC}}$ occur at the higher frequency mode. However, when the ratio cross through the transition point, $L_{x} /L > R_{t}$, the $c_{1}^{z} {\text{ } - \text{ BIC}}$ appear at the lower frequency mode. When $L_{x} /L = 4.4$, two exponents $\alpha_{1}^{N} = 2$ and $\alpha_{1}^{P} = 1.8$.

**Figure 6:**
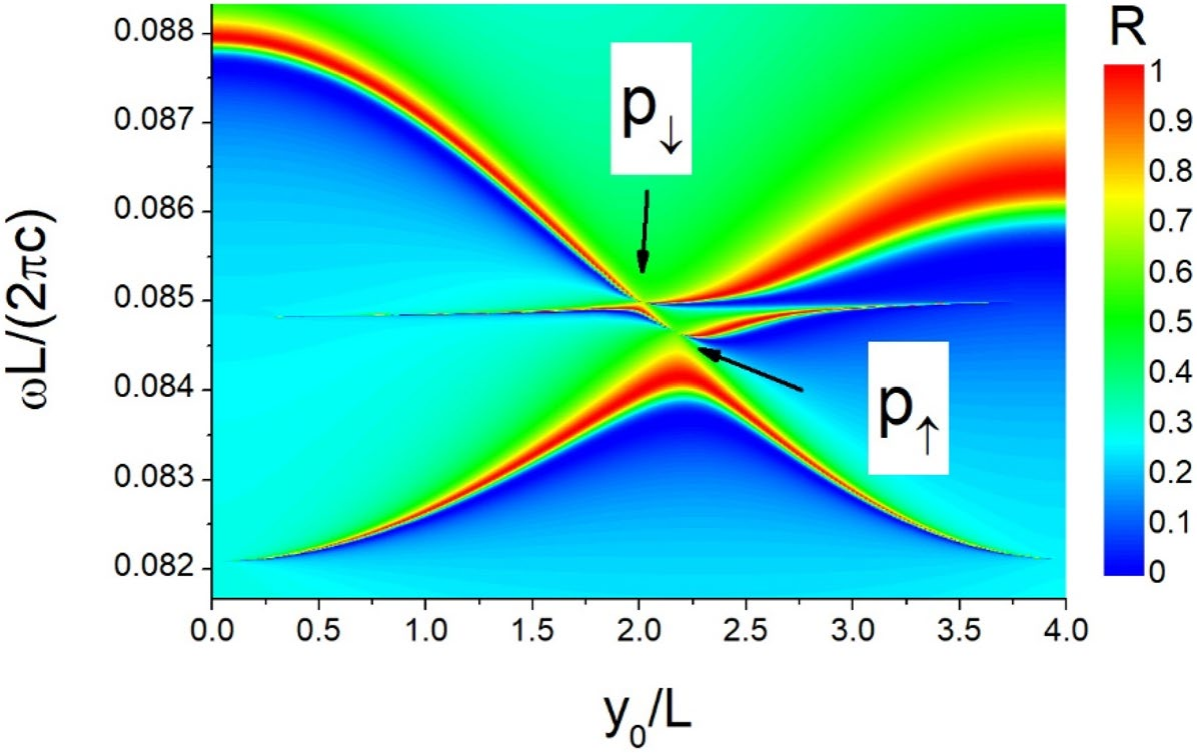
Reflectivity spectra of normal incident light as a function of frequency and displacement for rectangle air-hole slabs.

**Figure 7:**
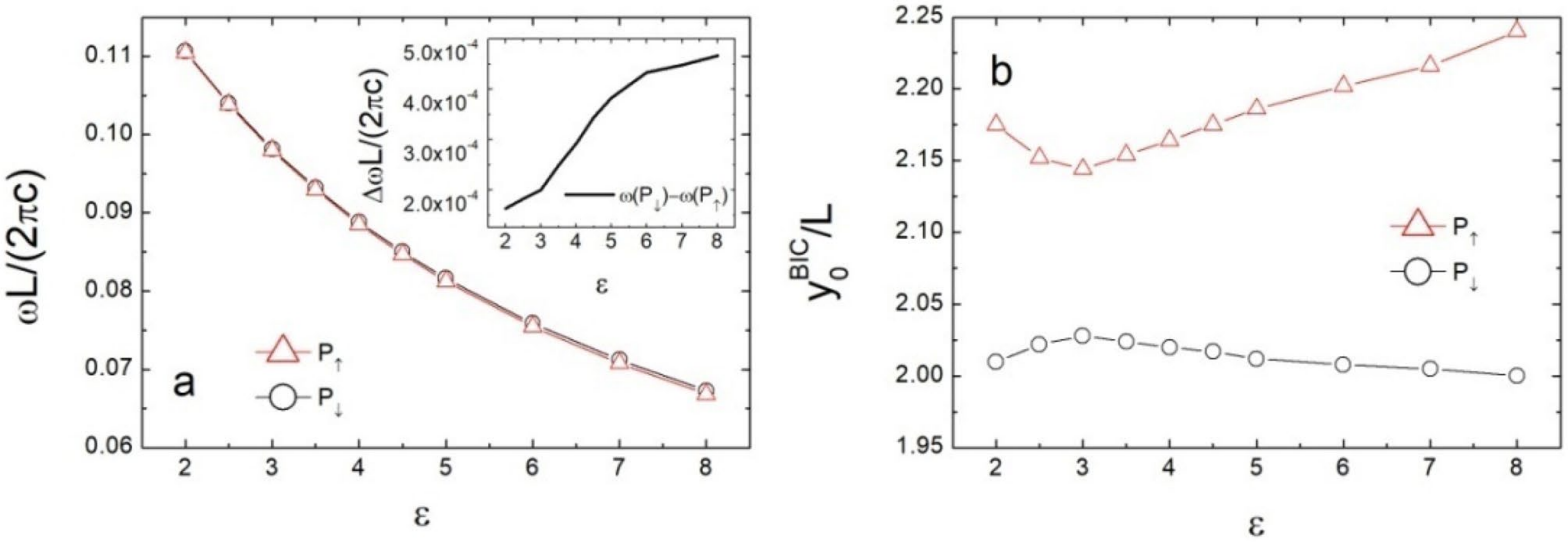
(**a**) Resonance frequency and (**b**) displacement of BIC as a function of the permittivity of slab.

Considering the PhC slab with rectangle air holes ($L_{y} /L = 2$ and $L_{x} /L = 4$), the reflectivity spectra of tri-layer structure are shown in Fig. 6. We can observe three modes and two $c_{1}^{z} {\text{ } - \text{ BICs}}$ guided by $P_{ \uparrow }$ and $P_{ \downarrow }$. By applying the Eq. (2), the resonance frequencies of $c_{1}^{z} {\text{ } - \text{ BICs}}$ could be estimated. When the permittivity of slab is increasing, the resonance frequencies of $c_{1}^{z} {\text{ } - \text{ BICs}}$ are decreasing as shown in Fig. 7a, but the difference between resonance frequencies is increasing. However, the displacements $y_{0}^{BIC} (P_{ \uparrow } )$ and $y_{0}^{BIC} (P_{ \downarrow } )$ have a non-monotonic function of $\varepsilon$ shown in Fig. 7b. These results illuminate that the waveguide modes in tri-layer structures can be adjusted by changing the permittivity of slab. Thus, the resonance frequencies of BIC can be manipulated by choosing various materials.

## Conclusion

In conclusions, we have demonstrated the formation of BICs in tri-layer systems made of three identical PhC slabs with square or rectangle air holes. The $c_{{2}}^{z}$ and $c_{{4}}^{z}$ types of BICs are accessible due to the symmetry incompatibility with the radiation. A distinct characteristic of the $c_{1}^{z} {\text{ } - \text{ BICs}}$ achieved in this study is that it originates from destructive interference of two resonances at the phase matching conditions. We introduce the two mode coupled model to obtain the formula to fit the reflectivity data and estimate the lifetime. The tri-layer structures, producing the various rotational symmetries, provide a new platform towards the BIC studies.

## Data Availability

The data that support the findings of this study are available from the corresponding author upon reasonable request.
